# Influence of contextualized physical performance metrics on offensive and defensive outcomes in professional football players

**DOI:** 10.5114/biolsport.2026.158681

**Published:** 2026-02-06

**Authors:** Paolo Troiani, Antonio Lucadamo, Dario Pompa, Cristian Savoia, Francesco Laterza, Maurizio Bertollo, Marco Beato

**Affiliations:** 1 Department of Medicine and Aging Sciences, University “G. d’Annunzio” of Chieti-Pescara, Chieti, Italy; 2 Department of Law, Economics, Management and Quantitative Methods (DEMM), University of Sannio, Benevento, Italy; 3 BIND-Behavioral Imaging and Neural Dynamics Center, University “G. d’Annunzio” of Chieti-Pescara, Chieti, Italy; 4 The Research Institute for Sport and Exercise Sciences, The Tom Reilly Building, Liverpool John Moores University, Liverpool, United Kingdom; 5 Department of Wellbeing, Nutrition and Sport, Pegaso Open University, Naples, Italy; 6 Department of Neurosciences, Biomedicine and Movement Sciences, University of Verona, Verona, Italy; 7 School of Health and Sports Sciences, University of Suffolk, Ipswich, United Kingdom

**Keywords:** Football (soccer), Match analysis, Training load, Kinematic, Video tracking

## Abstract

This study aimed to determine how contextualized physical performance metrics influence offensive and defensive outcomes in professional football. We examined external load and high-intensity actions over two seasons, adopting a dual-team approach including a reference team and its opponent. Positional data from all outfield players in 760 matches were collected using a video-based tracking system, capturing sprinting, accelerations, decelerations, and high-intensity efforts across in-possession, out-of-possession, and out-of-play phases. Sprinting distance in possession was associated with a higher number of goals scored (β = 0.308, standard error [SE] = 0.057, p < 0.001). High metabolic power distance during out-of-play phases was also a positive predictor of offensive effectiveness (β = 1.274, SE = 0.218, p < 0.001). High-intensity deceleration distance in possession was negatively related to goals scored (β = -0.257, SE = 0.089, p = 0.004). Defensively, higher opponent intensity—such as sprinting distance in possession and high metabolic power distance during out-of-play phases—was associated with a higher number of goals conceded (β = 0.212, SE = 0.052, p < 0.001; β = 1.379, SE = 0.207, p < 0.001, respectively), whereas high metabolic power distance of the reference team during out-of-play phases was negatively associated (β = -0.966, SE = 0.241, p < 0.001). The influence of in-possession decelerations differed between seasons, indicating that timing and tactical context modulate how these actions affect goals conceded. A focused set of contextualized physical metrics critically shape offensive and defensive outcomes and can guide training and tactical strategies to enhance team performance.

## INTRODUCTION

In recent years, the integration of performance analytics into professional football (soccer) has transformed how teams prepare, compete, and evaluate success [1, 2, 3]. Among the most widely adopted parameters in this evolving field of performance analysis are external training load metrics, which offer objective and quantifiable insights into the physical demands placed on players during both training sessions and competitions [1]. These data, collected primarily through GPS and video tracking systems, have become essential for monitoring training loads, managing training intensity and volume, and thus optimizing performance [2].

While total distance covered remains the most common kinematic parameter, it provides limited information about the neuromuscular and power-related demands associated with high-intensity actions such as accelerations, decelerations, and sprints. As a result, attention has increasingly shifted toward high-speed running (HSR) and sprinting distances (VHSR), typically defined as running above 19.8 km · h^−1^ and 25.2 km · h^−1^, respectively [4]. These variables are now widely recognized as key indicators of high game intensity [4, 5]. When examined across distinct phases of play such as inpossession and out-of-possession, and during tactically moments including pressing, counterattacking, and defensive transitions—these metrics yield critical insights into the physical demands associated with specific tactical behaviours [6]. This contextualized approach allows practitioners to better understand how physical exertion aligns with strategic intentions and contributes to overall team performance [3, 7]. Complementing these speed-based metrics, metabolic power has emerged as a valuable indicator of physical exertion [8, 9]. It indirectly estimates the energy cost associated with accelerations, decelerations, changes of direction and high-intensity activities, which are all actions common in football but not fully captured by traditional speed parameters [10]. High metabolic power thresholds (e.g., > 25.5 W · kg^−1^) and high-intensity acceleration loads (e.g., > 3.0 m · s^−2^) can provide another perspective of the metabolic demands placed on players during explosive efforts, which are central to both offensive and defensive performance [11].

Some recent studies have emphasized that such high-intensity metrics must be interpreted within their tactical context [12, 13]. Morgans et al. [6] demonstrated how in-possession and out-of-possession HSR and sprinting vary significantly by playing position, opponent quality and match location. Their findings show that physical performance varies substantially depending on tactical elements such as whether a team is in or out of possession, the quality of the opponent, and match location. This highlights the need to interpret physical metrics within the context of match strategy, rather than as isolated indicators of effort. Similarly, Prieto-González et al. [14] emphasized the relevance of defensive performance indicators, such as opponent ball touches and shots conceded, in explaining goals conceded in La Liga, highlighting the integrative value of combining physical and tactical datasets to elucidate match outcomes. From a physical standpoint, their analysis demonstrated that elevated defensive physical outputs, quantified through variables such as total distance covered, high-intensity sprints, and accelerations during defensive phases, are significantly associated with a lower incidence of goals conceded, underscoring the critical contribution of physical exertion to effective defensive performance [14]. Although interest in performance analytics continues to rise, there remains a scarcity of research on the physical-tactical dynamics within professional football. Elite-level competition offers a distinctive context for exploring the relationship between high-intensity physical performance and match outcomes, particularly in terms of goals scored and conceded, as playing style and tactical approaches vary substantially across teams and culture, reflecting the interplay between tactical traditions, coaching philosophies, and the evolving physical demands of the modern game [15, 16]. This gap is especially evident when considering the role of contextual factors such as opponent characteristics, tactical strategies, and match dynamics, which are essential for a nuanced understanding of performance in competitive settings.

Although previous studies have advanced our understanding of physical performance in football, most have focused on single-team analyses or descriptive metrics without accounting for tactical context or the interactive influence of the opponent. Consequently, there is limited knowledge on how high-intensity physical actions from both teams collectively influence match outcomes such as goals scored and conceded.

This study aims to extend previous work by Savoia et al. [17], who analysed the physical demands of professional football players using the K-Sport tracking system, by investigating how external load variables relate to match outcomes over two full seasons (2022/2023 and 2023/2024). While prior research has largely focused on single-team analyses or general descriptive metrics, the current paper addresses a critical gap by adopting a dual-team approach that incorporates physical performance data from both the reference team and its opponent. This relational framework captures the inherently interactive nature of elite football and allows for a more context-sensitive and ecologically valid assessment of physical performance in competition. In line with this objective, we hypothesize that greater external load values, particularly those associated with high-intensity actions, are positively associated with successful match outcomes, and that these associations vary depending on the opponent’s physical and tactical characteristics.

## MATERIALS AND METHODS

### Participants

This retrospective observational cohort study included all matches (*n* = 760) from professional football players during the seasons 2022/2023 and 2023/2024. A total of 23 teams were analysed. Data were collected for all outfield players from both teams in each match, allowing a dual-team approach that considers the performance of the reference team in relation to its opponent. Goalkeepers were excluded from the analysis. This approach enables evaluation of physical performance within the interactive context of competitive football. Due to regulatory constraints within the championship, which limit the number of participating teams, we employed a convenient sampling approach; no additional teams could be included beyond those permitted by league rules. Furthermore, a priori power analysis was not performed given the exceptionally large dimensions of the sample. The substantial sample size ensures high statistical power, rendering the likelihood of Type II error limited. Players’ data were collected as part of routine performance monitoring conducted by the club during official matches. No experimental intervention was introduced, and the data collection procedures did not modify players’ standard training or match routines, nor did they influence their physical load in any way. As the monitoring formed part of the organization’s established operational practices and did not involve any manipulation of behaviour or performance, formal ethical approval from an institutional review board was not required. All personal and team-related information was fully anonymized prior to analysis [18]. Nonetheless, all personal and team-specific data were anonymized prior to analysis. The study was conducted in accordance with the ethical principles outlined in the Declaration of Helsinki, thereby safeguarding the confidentiality and welfare of all participating individuals.

### Data collection

Match data were collected using the Hawk-Eye system (Hawk-Eye Innovations Limited, Basingstoke, UK). The Hawk-Eye workflow typically includes triangulation-based positional estimation, video calibration, and post-processing through delayed filtering, in order to maximize positional accuracy after the game [19]. Tracking information was processed using Dynamix (K-Sport World S.R.L., Pesaro, Italy; software version 1.25.6), which serves as the main platform for acquiring and standardizing the tracking data inputs [17]. Variables were categorized according to team identity (reference vs. opponent) and phase of play, defined as in-possession (team in control of the ball), out-of-possession (team not in control of the ball), or out-of-play (ball not in play). Following thresholds commonly applied in elite football research [7], the analysis included sprinting distance (VHSR, > 25.2 km · h^−1^), average metabolic power (W · kg^−1^), high metabolic power distance (HMPD, > 20 W · kg^−1^), as well as high-intensity acceleration distance (H-acc, > 3 m · s^−2^) and deceleration distance (Hdec, < –3 m · s^−2^). Metabolic power was estimated using the model developed by Osgnach et al. [10], which derives instantaneous energy cost by treating accelerated running as equivalent to uphill running at an “equivalent slope”, adjusted for the higher cost of running on grass; multiplying this cost by running speed yields the instantaneous metabolic power. These indicators were extracted separately for each of the three phases of play, except for high-speed running distance (HSR, 19.8–25.2 km · h^−1^), which was considered only in aggregate form. This framework was designed to capture the contextualized contribution of high-intensity efforts most relevant to offensive and defensive outcomes. A complete overview of the variables and their operational definitions is reported in Table 1.

**TABLE 1:** Variables description for the reference team and opponent

Variable name	Description
VHSR_WB_	Mean distance covered in sprinting actions (> 25.2 km · h^−1^) in-possession
VHSR_NB_	Mean distance covered in sprinting actions (> 25.2 km · h^−1^) out-of-possession
VHSR_OP_	Mean distance covered in sprinting actions (> 25.2 km · h^−1^) during out-of-play phases
HSR	Mean distance covered at a running speed between 19.8 and 25.2 km · h^−1^
AMP_WB_	Average metabolic power (W · kg^−1^) in-possession
AMP_NB_	Average metabolic power (W · kg^−1^) out-of-possession
HMPD_WB_	Mean distance covered at > 20 W · kg^−1^ metabolic power in-possession
HMPD_NB_	Mean distance covered at > 20 W · kg^−1^ metabolic power out-of-possession
HMPD_OP_	Mean distance covered at > 20 W · kg^−1^ metabolic power during out-of-play phases
H-dec_WB_	Mean distance covered in decelerations < -3 m · s^−2^ in-possession
H-dec_NB_	Mean distance covered in decelerations < -3 m · s^−2^ out-of-possession
H-dec_OP_	Mean distance covered in decelerations < -3 m · s^−2^ during out-of-play phases
H-acc_WB_	Mean distance covered in accelerations > 3 m · s^−2^ in-possession
H-acc_NB_	Mean distance covered in accelerations > 3 m · s^−2^ out-of-possession
H-acc_OP_	Mean distance covered in accelerations > 3 m · s^−2^ during out-of-play phases

All performance indicators were analysed at the match level, computed as weighted averages of the players who participated, with weights corresponding to minutes played. This approach accounts for individual contributions without treating all players’ data uniformly at the team level. No special handling was applied for matches in which a team played with ten players following a red card. All player contributions were averaged based on minutes played, regardless of in-game dismissals. We acknowledge that playing with a reduced number of players can influence physical output and match dynamics, and this represents a valid consideration for future analyses.

### Statistical analysis

Statistical analyses were conducted using R software (version 4.4.3; R Core Team, 2025, R Foundation for Statistical Computing, Vienna, Austria). The response variables—the number of goals scored and conceded—were discrete, non-negative counts. They were therefore first examined using correlation analyses (Pearson’s r, Spearman’s, and Kendall’s) to assess the relationship between the two outcomes. Although these analyses indicated a modest association, the dependence was not strong enough to justify a joint modelling approach. Consequently, and to isolate the effects of covariates on each outcome independently, the responses were modelled separately, while acknowledging that future studies could adopt multivariate frameworks.

Analyses were conducted separately for the 2022/23 and 2023/24 seasons to account for potential season-specific variations in team composition, training schedules, and match contexts. Presenting the results separately allows for a more accurate assessment of performance indicators within each season and avoids potential confounding effects that could arise if data were pooled.

Given the count-based nature of the outcomes, generalized linear modelling with a Poisson distribution was considered appropriate. Poisson regression is widely used in sports analytics for modelling event counts, as it naturally accounts for the integer-valued structure of the data and their typically right-skewed distribution. A methodological challenge in this context is the potential multicollinearity among explanatory variables, since physical performance indicators often co-vary in competitive match settings. Additionally, the relatively high number of covariates increases the risk of overfitting. To mitigate these issues, a regularized Poisson regression with LASSO penalty was employed [20, 21]. LASSO (Least Absolute Shrinkage and Selection Operator) applies an L1 penalty to the regression coefficients, shrinking less informative predictors toward zero. This produces a more parsimonious model, enhances interpretability, and improves predictive performance by performing variable selection. Compared to ridge regression, which shrinks coefficients continuously but does not eliminate variables, LASSO was selected for its ability to isolate the most influential performance metrics. To account for the relational dynamics of match play, covariates from both the reference team and the opposing team were included. Parameter estimation was achieved by minimizing the penalized log-likelihood function [22]:

$$-\frac{1}{N}\sum_{i=1}^{n}\left(y_{i}(\beta_{0}+\beta^{T}x_{i})-e^{\beta 0+\beta^{T}}x_{i}\right)+\lambda (\alpha \sum_{i=1}^{n}|\beta_{i}|)$$

where *y_i_* denotes the observed outcome for match *i, X*_*i*_ represents the vector of covariates, and β is the corresponding regression coefficient. The penalty term λ controls the degree of shrinkage (determined through cross-validation), while α specifies the contribution of the L1 component (in this study, α was set to 1 to implement pure LASSO regularization). This analytical framework allowed the study to identify which contextualized physical performance metrics, across both teams and match phases, most strongly influenced offensive and defensive outcomes, providing an evidence-based foundation for understanding match dynamics in elite football. Statistical significance was set at p < 0.05.

## RESULTS

Across the 2022/23 and 2023/24 seasons, several contextualized physical performance indicators were significantly associated with match outcomes (see Tables 2–5).

**TABLE 2:** Estimates from the Poisson regression model after LASSO selection for scored goals (2022/23)

Variable	Estimate	Std.error	Statistic	p.value	signif
(Intercept)	0.237	0.048	4.958	< 0.001	***
**Reference Team**					
VHSR_WB_	0.306	0.081	3.784	< 0.001	***
VHSR_OP_	0.127	0.171	0.740	0.459	
HSR	-0.130	0.076	−1.700	0.089	
AMP_WB_	-0.027	0.074	−0.370	0.712	
AMP_NB_	0.032	0.097	0.330	0.741	
HMPD_WB_	-0.273	0.176	−1.545	0.122	
HMPD_OP_	1.365	0.344	3.963	< 0.001	***
H-dec_WB_	-0.274	0.132	−2.067	0.039	*
H-dec_NB_	0.301	0.145	2.079	0.038	*
H-dec_OP_	-0.899	0.227	−3.963	< 0.001	***
H-acc_WB_	0.413	0.141	2.923	0.003	**
H-acc_NB_	−0.371	0.155	−2.397	0.016	*
H-acc_OP_	0.667	0.207	3.216	0.001	**

**Opponent**					
VHSR_WB_	−0.098	0.067	−1.453	0.146	
VHSR_NB_	−0.058	0.080	−0.729	0.466	
VHSR_OP_	−0.196	0.177	−1.108	0.268	
HSR	0.200	0.092	2.181	0.029	*
AMP_WB_	0.133	0.100	1.332	0.183	
HMPD_NB_	−0.040	0.177	−0.224	0.823	
HMPD_OP_	−1.105	0.347	−3.179	0.001	**
H-dec_WB_	−0.258	0.136	−1.902	0.057	
H-dec_OP_	0.751	0.246	3.055	0.002	**
H-acc_WB_	0.261	0.148	1.764	0.078	
H-acc_NB_	−0.171	0.114	−1.498	0.134	
H-acc_OP_	−0.777	0.220	−3.527	< 0.001	***

*** *p* < 0.001;

** *p* < 0.01;

* *p* < 0.05

VHSR_WB_: sprinting distance in-possession; VHSR_NB_: sprinting distance out-of-possession; VHSR_OP_: sprinting distance out-of-play; HSR: high-speed running distance; AMP_WB_: average metabolic power in-possession; AMP_NB_: average metabolic power out-of-possession; HMPD_WB_: high metabolic power distance in-possession; HMPD_NB_: high metabolic power distance out-of-possession; HMPD_OP_: high metabolic power distance out-of-play; H-dec_WB_: high-intensity deceleration distance in-possession; H-dec_NB_: high-intensity deceleration distance out-of-possession; H-dec_OP_: high-intensity deceleration distance out-of-play; H-acc_WB_: high-intensity acceleration distance inpossession; H-acc_NB_: high-intensity acceleration distance out-of-possession; H-acc_OP_: high-intensity acceleration distance out-of-play.

**TABLE 3:** Estimates from the Poisson regression model after LASSO selection for conceded goals (2022/23)

Variable	Estimate	Std.error	Statistic	p.value	signif
(Intercept)	0.017	0.054	0.318	0.751	
**Reference Team**					
VHSR_WB_	-0.208	0.092	-2.251	0.024	*
VHSR_NB_	0.104	0.092	1.133	0.257	
VHSR_OP_	-0.226	0.195	-1.159	0.246	
HSR	0.308	0.102	3.008	0.003	**
AMP_WB_	0.242	0.076	3.174	0.001	**
AMP_NB_	0.414	0.232	1.784	0.074	
HMPD_NB_	-0.530	0.251	-2.115	0.034	*
HMPD_OP_	-1.215	0.373	-3.252	0.001	**
H-dec_WB_	-0.413	0.161	-2.556	0.011	*
H-dec_NB_	0.219	0.177	1.237	0.216	
H-dec_OP_	0.155	0.159	0.983	0.325	
H-acc_WB_	0.218	0.167	1.306	0.192	
H-acc_NB_	-0.172	0.164	-1.049	0.294	
H-acc_OP_	-0.255	0.145	-1.753	0.080	

**Opponent**					
VHSR_WB_	0.208	0.101	2.066	0.039	*
VHSR_NB_	-0.037	0.097	-0.381	0.703	
VHSR_OP_	-0.278	0.198	-1.405	0.160	
HSR	-0.239	0.112	-2.134	0.033	*
AMP_WB_	-0.345	0.196	-1.762	0.078	
HMPD_WB_	0.145	0.239	0.606	0.544	
HMPD_NB_	0.146	0.146	1.000	0.317	
HMPD_OP_	1.816	0.357	5.092	< 0.001	***
H-dec_WB_	-0.212	0.161	-1.322	0.186	
H-dec_NB_	0.182	0.189	0.965	0.334	
H-acc_WB_	0.286	0.154	1.858	0.063	
H-acc_NB_	-0.048	0.178	-0.270	0.787	

*** *p* < 0.001;

** *p* < 0.01;

* *p* < 0.05

VHSR_WB_: sprinting distance in-possession; VHSR_NB_: sprinting distance out-of-possession; VHSR_OP_: sprinting distance out-of-play; HSR: high-speed running distance; AMP_WB_: average metabolic power in-possession; AMP_NB_: average metabolic power out-of-possession; HMPD_WB_: high metabolic power distance in-possession; HMPD_NB_: high metabolic power distance out-of-possession; HMPD_OP_: high metabolic power distance out-of-play; H-dec_WB_: high-intensity deceleration distance in-possession; H-dec_NB_: high-intensity deceleration distance out-of-possession; H-dec_OP_: high-intensity deceleration distance out-of-play; H-acc_WB_: high-intensity acceleration distance inpossession; H-acc_NB_: high-intensity acceleration distance out-of-possession; H-acc_OP_: high-intensity acceleration distance out-of-play.

**TABLE 4:** Estimates from the Poisson regression model after LASSO selection for scored goals (2023/24)

Variable	Estimate	Std.error	Statistic	p.value	signif
(Intercept)	0.263	0.047	5.588	< 0.001	***
**Reference Team**					
VHSR_WB_	0.309	0.081	3.833	< 0.001	***
VHSR_NB_	−0.003	0.086	−0.040	0.968	
HSR	−0.321	0.111	−2.891	0.004	**
AMP_WB_	−0.135	0.126	−1.070	0.285	
AMP_NB_	0.259	0.068	3.791	< 0.001	***
HMPD_NB_	0.027	0.171	0.157	0.875	
HMPD_OP_	1.212	0.282	4.298	< 0.001	***
H-dec_WB_	−0.244	0.119	−2.044	0.041	*
H-dec_NB_	−0.176	0.141	−1.246	0.213	
H-dec_OP_	−0.187	0.119	−1.569	0.117	
H-acc_NB_	0.131	0.132	0.993	0.321	
H-acc_OP_	0.126	0.182	0.693	0.488	

**Opponent**					
VHSR_WB_	−0.090	0.088	−1.012	0.311	
VHSR_NB_	−0.157	0.082	−1.922	0.055	
VHSR_OP_	−0.071	0.105	−0.676	0.499	
HSR	0.244	0.120	2.043	0.041	*
AMP_NB_	−0.066	0.137	−0.481	0.630	
HMPD_WB_	−0.132	0.156	−0.849	0.396	
HMPD_NB_	0.120	0.159	0.754	0.451	
HMPD_OP_	−0.811	0.267	−3.039	0.002	**
H-acc_WB_	0.191	0.132	1.441	0.150	
H-acc_NB_	−0.051	0.111	−0.457	0.648	
H-acc_OP_	−0.240	0.183	−1.314	0.189	

*** *p* < 0.001;

** *p* < 0.01;

* *p* < 0.05

VHSR_WB_: sprinting distance in-possession; VHSR_NB_: sprinting distance out-of-possession; VHSR_OP_: sprinting distance out-of-play; HSR: high-speed running distance; AMP_WB_: average metabolic power in-possession; AMP_NB_: average metabolic power out-of-possession; HMPD_WB_: high metabolic power distance in-possession; HMPD_NB_: high metabolic power distance out-of-possession; HMPD_OP_: high metabolic power distance out-of-play; H-dec_WB_: high-intensity deceleration distance in-possession; H-dec_NB_: high-intensity deceleration distance out-of-possession; H-dec_OP_: high-intensity deceleration distance out-of-play; H-acc_WB_: high-intensity acceleration distance inpossession; H-acc_NB_: high-intensity acceleration distance out-of-possession; H-acc_OP_: high-intensity acceleration distance out-of-play.

**TABLE 5:** Estimates from the Poisson regression model after LASSO selection for conceded goals (2023/24)

Variable	Estimate	Std.error	Statistic	p.value	signif
(Intercept)	0.059	0.052	1.133	0.257
**Reference Team**					
VHSR_WB_	−0.160	0.097	−1.645	0.100	
VHSR_OP_	−0.062	0.131	−0.472	0.637	
HSR	0.090	0.101	0.886	0.376	
AMP_WB_	0.016	0.137	0.114	0.909	
HMPD_NB_	−0.019	0.144	−0.130	0.896	
HMPD_OP_	−0.789	0.316	−2.498	0.012	*
H-dec_WB_	0.309	0.139	2.218	0.027	*
H-dec_NB_	0.127	0.166	0.761	0.447	
H-acc_NB_	−0.006	0.138	−0.047	0.963	
H-acc_OP_	−0.130	0.144	−0.901	0.367	

**Opponent**					
VHSR_WB_	0.214	0.061	3.493	< 0.001	***
VHSR_NB_	−0.014	0.093	−0.155	0.876	
AMP_WB_	−0.072	0.064	−1.117	0.264	
AMP_NB_	0.138	0.155	0.890	0.374	
HMPD_NB_	−0.287	0.154	−1.858	0.063	
HMPD_OP_	1.158	0.254	4.567	< 0.001	***
H-dec_WB_	−0.380	0.143	−2.654	0.008	**
H-dec_NB_	−0.084	0.145	−0.582	0.560	
H-dec_OP_	−0.194	0.135	−1.440	0.150	
H-acc_WB_	0.155	0.137	1.130	0.259	

*** *p* < 0.001;

** *p* < 0.01;

* *p* < 0.05

VHSR_WB_: sprinting distance in-possession; VHSR_NB_: sprinting distance out-of-possession; VHSR_OP_: sprinting distance out-of-play; HSR: high-speed running distance; AMP_WB_: average metabolic power in-possession; AMP_NB_: average metabolic power out-of-possession; HMPD_NB_: high metabolic power distance out-of-possession; HMPD_OP_: high metabolic power distance out-of-play; H-dec_WB_: highintensity deceleration distance in-possession; H-dec_NB_: high-intensity deceleration distance out-of-possession; H-dec_OP_: high-intensity deceleration distance out-of-play; H-acc_WB_: high-intensity acceleration distance in-possession; H-acc_NB_: high-intensity acceleration distance out-of-possession; H-acc_OP_: high-intensity acceleration distance out-of-play.

**FIG. 1:**
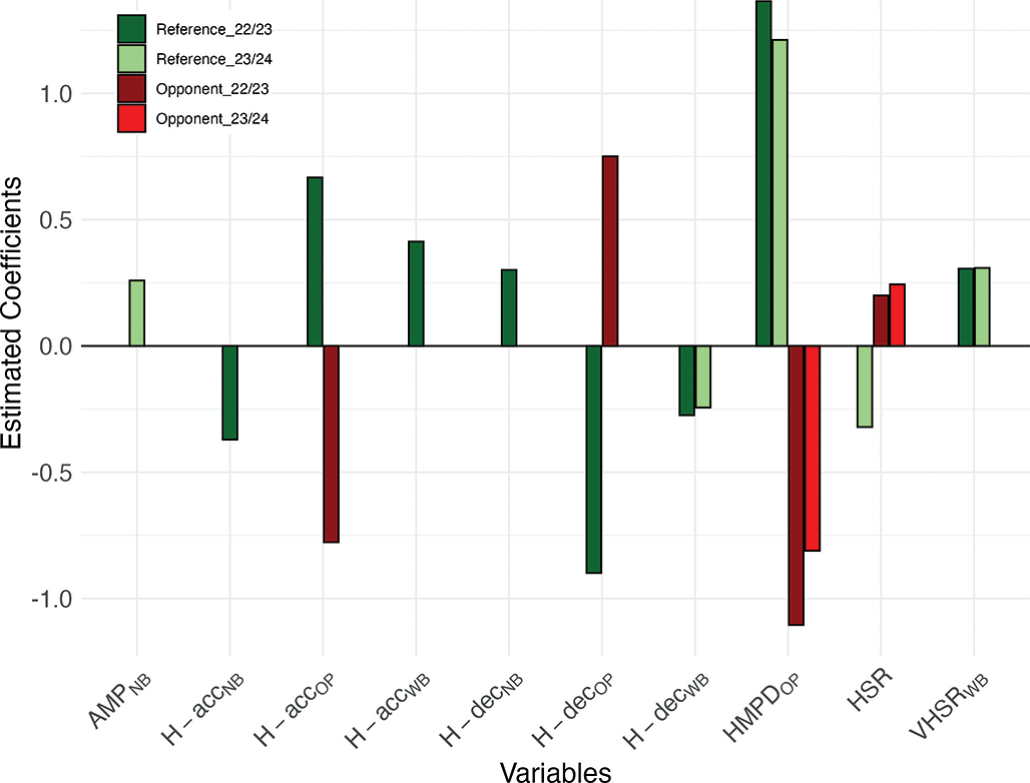
Significant predictors of goals scored across both seasons (2022/23 and 2023/24). x-axis shows variables, y-axis shows estimated coefficients (β) from a Poisson model fitted on standardized variables. Coefficients (β) indicate how a one–SD increase in each predictor changes the log expected scoring rate; exp β gives the multiplicative effect on the expected goal-scoring rate (values > 1 indicate an increase, values < 1 a decrease).

For goals scored, reference team variables such as sprinting distance in possession (VHSR_WB_) and high metabolic power distance during out-of-play phases (HMPD_OP_) consistently showed positive associations across both seasons (VHSR_WB_: 2022/23: β = 0.306, SE = 0.081, p < 0.001; 2023/24: β = 0.309, SE = 0.081, p < 0.001; HMPD_OP_: 2022/23: β = 1.365, SE = 0.344, p < 0.001; 2023/24: β = 1.212, SE = 0.282, p < 0.001), while high-intensity deceleration distance in possession (H-dec_WB_) was negatively associated (2022/23: β = −0.274, SE = 0.132, p = 0.039; 2023/24: β = −0.244, SE = 0.119, p = 0.041) (Tables 2 and 4). Other reference team indicators with season-specific effects included high-intensity acceleration distance in possession (H-acc_WB_) (Table 2), high-intensity acceleration distance during out-of-play phases (H-acc_OP_) (Table 2), high-intensity deceleration distance out of possession (H-dec_NB_) (Table 2), average metabolic power out of possession (AMP_NB_) (Table 4), and high-speed running distance (HSR) (Table 4). In addition, opponent-related variables such as HMPD_OP_, high-intensity deceleration distance during out-of-play phases (H-dec_OP_), and H-acc_OP_ also showed significant associations with goals scored, although their effects varied depending on the season (Tables 2 and 4). The significant predictors of goals scored across both seasons are visually summarized in Figure 1.

For goals conceded, the reference team variable HMPD_OP_ consistently showed a negative association across both seasons (2022/23: β = −1.215, SE = 0.373, p = 0.001; 2023/24: β = −0.789, SE = 0.316, p = 0.012), indicating a protective effect, while H-dec_WB_ displayed contrasting associations depending on the season (2022/23: β = −0.413, SE = 0.161, p = 0.011; 2023/24: β = 0.309, SE = 0.139, p = 0.027) (Tables 3 and 5).

**FIG. 2:**
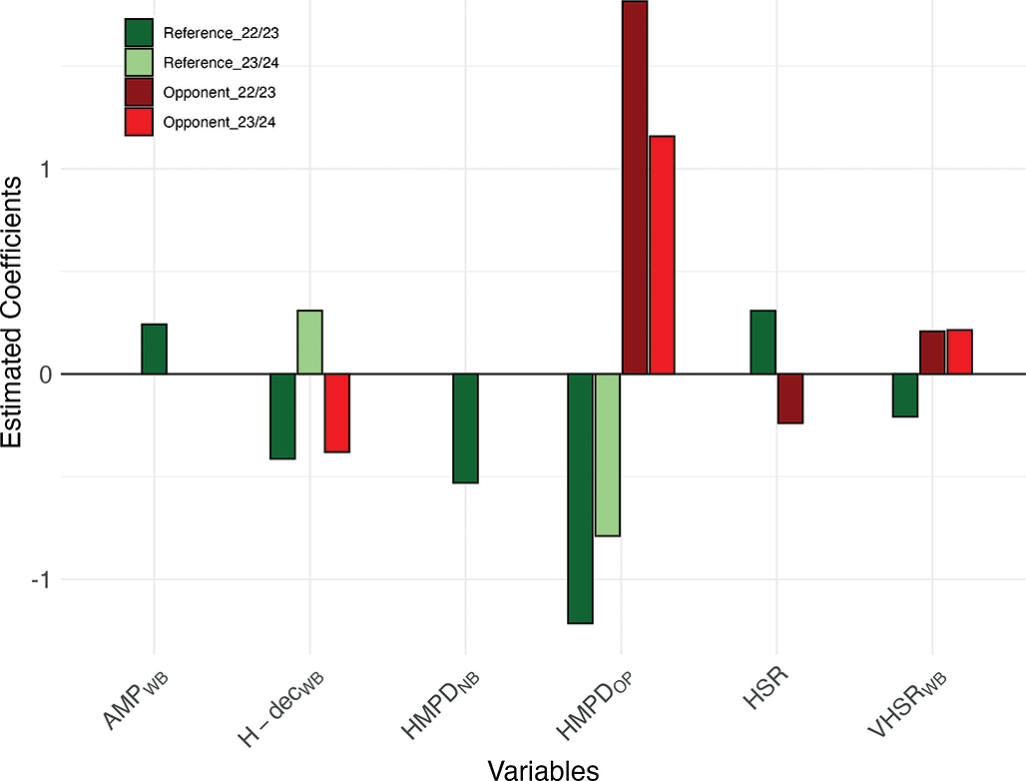
Significant predictors of goals conceded across both seasons (2022/23 and 2023/24). x-axis shows variables, y-axis shows estimated coefficients (β) from a Poisson model fitted on standardized variables. Coefficients (β) indicate how a one–SD increase in each predictor changes the log expected rate of goals conceded; exp β gives the multiplicative effect on the expected number of goals conceded (values > 1 indicate an increase, values < 1 a decrease).s

Opponent-related variables strongly predicted defensive vulnerability, with VHSR_WB_ and HMPD_OP_ positively associated with goals conceded across both seasons. Specifically, VHSR_WB_ showed positive associations in both 2022/23 (β = 0.208, SE = 0.101, p = 0.039) and 2023/24 (β = 0.214, SE = 0.061, p < 0.001), whereas HMPD_OP_ displayed positive effects in both 2022/23 (β = 1.816, SE = 0.357, p < 0.001) and 2023/24 (β = 1.158, SE = 0.254, p < 0.001) (Tables 3 and 5). By contrast, H-dec_WB_ from the opponent team demonstrated protective effects in certain contexts, reducing the likelihood of conceding (Tables 3 and 5). Figure 2 provides a visual summary of the significant predictors of goals conceded across both seasons.

Overall, several variables emerged as consistent predictors across both seasons. For the reference team, VHSR_WB_ and HMPD_OP_ were positively associated with goals scored, whereas for the opponent team, both variables increased the risk of conceding goals.

## DISCUSSION

This study investigated the relationship between external load metrics and match outcomes across the 2022/23 and 2023/24 seasons, employing a dual-team, context-sensitive framework [23]. We hypothesized that elevated external load values—especially those reflecting high-intensity actions—would be positively associated with successful performance, and that these associations would vary according to the physical and tactical profiles of opposing teams. Our findings highlight sprinting distance (VHSR) and high metabolic power distance (HMPD) as critical predictors of both offensive and defensive success. Consequently, the identified importance of VHSR and HMPD aligns with broader evidence that both high-intensity locomotor actions and technical possession indicators jointly drive competitive performance in elite European football [24, 25]. Thus, integrating zone-specific positioning strategies with calibrated high-intensity training may further amplify both offensive creation and defensive solidity [26, 27].

Scoring success was positively associated with sprinting distance during in-possession phases. Conversely, conceding goals was more likely with greater opponent activity, particularly during out-of-possession phases. This suggests that opponent intensity may reflect pressing efficiency or sustained defensive pressure that amplifies offensive threat. Moreover, performance measures during out-of-play moments, when the ball is stationary, also showed predictive relevance, possibly indicating levels of fatigue, recovery, or team readiness. Overall, these findings highlight the importance of integrating contextualized physical metrics with tactical insights to advance the understanding of the demands of elite football match play. Nevertheless, it is important to note that some studies suggest that technical and tactical factors may be more predictive than physical parameters, particularly in top-tier competitions such as La Liga, whereas high-speed running distance (HSR) appeared more relevant in the Spanish second division [28].

An additional factor that may modulate the associations observed in the present study is the scheduling density typical of elite football. During congested periods — characterised by ≤ 3–4 days between matches — training content and weekly load distribution are necessarily adjusted to prioritise recovery. Recent evidence shows that shorter microcycles markedly reduce high-speed running, sprinting exposure, and the number of accelerations and decelerations performed in training, where coaches tend to minimise neuromuscular load to preserve readiness for competition [29]. Consequently, players may enter matches with altered preparation profiles, potentially influencing high-intensity actions and their tactical expression. Moreover, studies conducted during dense fixture periods in top-level teams demonstrate that starters accumulate substantially greater external and internal loads than non-starters, including higher total distance, rating of perceived exertion (RPE), and individual very highspeed running relative to maximal velocity [30]. This imbalance may exacerbate fatigue across successive matches and affect both the physical metrics quantified in this study and their relationship with goal-related outcomes. While the present analysis included two full seasons and therefore inherently captured periods of varying schedule density, future research integrating microcycle length or match congestion as explicit contextual variables could clarify how weekly load constraints shape the execution, timing, and effectiveness of high-intensity actions.

For goals scored, a consistent relationship emerged with high-intensity actions performed by the reference team during possession phases. Among these, sprinting distance in possession (VHSR_WB_) was the most prominent predictor, showing a positive association across both seasons (2022/23: p < 0.001; 2023/24: p < 0.001). Our results highlight the importance of sprinting distance in possession, adding new insight to previous literature showing that straight-line sprints are the most frequent actions preceding goals [31]. This finding supports previous evidence highlighting the decisive role of direct, high-speed ball-carrying runs in creating scoring opportunities [5, 6, 32, 33]. In line with this perspective, Allen et al. [34] reported significant correlations between HSR, VHSR, and final league position in the English Premier League, noting that winning teams typically covered greater running distance compared to losing teams [34]. Similar evidence was reported by the study of Makar et al. [35], where, in the Turkish Super League, the number of accelerations performed within the 5.5–7.0 m/s range (i.e., accelerations occurring during HSR) significantly predicted team success. Notably, these accelerations occur just below the sprint threshold and, by definition, contribute to increasing players’ velocity. This suggests that the ability to frequently accelerate at high speeds, and subsequently sustain sprinting efforts, may represent a decisive factor in performance outcomes. In addition, Savoia et al. [17] demonstrated that high-intensity accelerations, defined as any event exceeding 50% of the player’s maximal acceleration capacity at a given initial speed, during possession phases, differentiated successful professional football teams. This approach accounts for accelerations initiated at different running speeds, appropriately weighting those occurring at higher velocities that are often overlooked by traditional, speed-independent thresholds. Taken together, these studies underline that offensive effectiveness depends not only on the presence of high-intensity actions but also on their contextual embedding within tactical structures and positional responsibilities.

When considering shots on goal, high-intensity activities have also been investigated as a potential discriminant factor, although the results have been mixed. Filetti et al. [36] reported that a greater proportion of successful shots on target were preceded by highintensity activities (in terms of runs > 21.6 km · h^−1^, accelerations > |3| m · s^−2^, and metabolic power > 55 W · kg^−1^) compared with unsuccessful attempts (71.1% vs 61.5%, χ^2^ test, p < 0.001. In contrast, Konefal et al. [37] found no significant differences in HSR or sprint distance in the five minutes preceding goals (∆ 95%CI -12.5 to 31.1 m; *p* = 0.401; ∆ 95%CI -17.3 to 26.3 m; *p* = 0.684). Moreover, high metabolic power distance during out-of-play phases (HMPD_OP_) was also positively associated/correlated with goals scored. This outcome may not directly reflect in-game tactical advantage but rather indicate enhanced physical readiness, recovery efficiency, and the ability to sustain high performance following stoppages [9]. Other strong contributors included high-intensity deceleration distance in possession (H-dec_WB_), which was negatively associated with goals scored. This is in line with previous observations showing that forward-directed actions—such as successful dribbles, runs with the ball, and long forward passes—led to significantly more scoring opportunities (and goals) than sideways or backwards play [38]. This indicates that phases when the team collectively slows down its offensive play can disrupt attacking momentum and limit opportunities to generate scoring chances.

**TABLE 6:** Consistent predictors of match outcomes across both 2022/23 and 2023/24 seasons

Variable	Team	Outcome	Direction
VHSR_WB_	Reference	Scored Goals	Positive
HMPD_OP_	Reference	Scored Goals	Positive
H-dec_WB_	Reference	Scored Goals	Negative
HMPD_OP_	Reference	Conceded Goals	Negative
HSR	Opponent	Scored Goals	Positive
HMPD_OP_	Opponent	Scored Goals	Negative
VHSR_WB_	Opponent	Conceded Goals	Positive
HMPD_OP_	Opponent	Conceded Goals	Positive

VHSR_WB_: sprinting distance in-possession; HMPD_OP_: high metabolic power distance out-of-play; H-dec_WB_: high-intensity deceleration distance in-possession; HSR: high-speed running distance). Scored goals: number of goals scored by the reference team; Conceded goals: number of goals the reference team allows. Positive: A one–SD increase in the variable is associated with an increase in the expected goal-scoring/conceding rate (exp(β) > 1); Negative: A one–SD increase in the variable is associated with a decrease in the expected goal-scoring/conceding rate (exp(β) < 1).

Beyond the variables consistent across both seasons, some predictors emerged reached significance in only one. In 2022/23, highintensity acceleration distance in-possession (H-acc_WB_) enhanced scoring opportunities, whereas high-intensity deceleration distance during out-of-play phases (H-dec_OP_) were linked to fewer goals.

In 2023/24, average metabolic power out-of-possession (AMP_NB_) supported goal creation, while HSR showed a negative association with offensive efficiency, likely reflecting suboptimal timing, execution or positioning of runs. This overall pattern reflects the broader tendency observed in elite football, where players perform a higher number of high-intensity actions without possession than with possession, especially in low-possession teams [3, 5, 17, 39]. Opponent dynamics also shaped offensive outcomes [40]. A notable example is HMPD_OP_, which was negatively associated with goals scored, suggesting that reduced opponent activity during stoppages may provide more favourable conditions for offensive buildup. Conversely, opponent HSR correlated positively with goals scored, potentially reflecting defensive disorganization that can be exploited by the attacking side. Season-specific variations further refined this picture: in 2022/23, higher high-intensity acceleration distance during outof-play phases (H-acc_OP_) was linked to fewer goals scored. Taken together, these findings highlight that offensive effectiveness depends not only on the execution of high-intensity actions but also on their timing, context and the behaviours of the opposition.

Shifting the focus to defensive vulnerability, several indicators emerged as consistent across both seasons. The reference team’s HMPD_OP_ displayed a negative association with goals conceded, indicating that maintaining physical readiness during stoppages can reduce defensive exposure once play resumes. At the same time, Hdec_WB_ showed a positive association with goals conceded, suggesting that hesitation or disruption in offensive transitions can increase the team’s susceptibility to breakdowns and counterattacks. Seasonspecific results also added nuance. In 2022/23, greater VHSR_WB_ reduced goals conceded by sustaining offensive momentum, whereas higher HSR was linked to defensive imbalances and more goals against. Opponent-related indicators consistently predicted defensive outcomes as well, underlining the bidirectional impact of physical intensity on match results. Across both seasons, opponent HMPD_OP_ showed a positive association with goals conceded, suggesting that continuous high-intensity pressing or recovery efforts by the opposition can compromise defensive stability. Similarly, greater VHSR_WB_ from opponents heightened the risk of conceding, emphasizing the threat posed by direct, rapid ball-carrying runs. Season-specific effects highlighted additional patterns: in 2023/24, higher opponent H-dec_WB_ coincided with fewer goals conceded, suggesting that periods of reduced opponent intensity eased defensive management; in 2022/23, higher opponent HSR also contributed to defensive protection by restricting attacking opportunities.

Overall, these findings indicate that defensive vulnerability depends not only on the team’s own outputs but also on the intensity, timing, and contextual characteristics of opponent actions. While some associations remained consistent across seasons, others reflected season-specific dynamics, highlighting the importance of integrating physical metrics within tactical and situational frameworks. Together with prior evidence [6, 17], our results reinforce that performance analysis in elite football requires a combined consideration of physical indicators, tactical execution, and opponent behaviour. This approach goes beyond traditional statistical analysis, providing a more refined understanding of match demands and equipping coaches and analysts with a solid foundation to develop strategies that enhance both offensive creation and defensive solidity [41].

### Study Limitations and Future Directions

While this study provides valuable insights into how context-specific physical metrics relate to goal dynamics, some limitations must be acknowledged. First, the observational design restricts causal inference. Although significant associations were identified between physical performance indicators and match outcomes, these relationships should not be interpreted as definitive evidence of causality. Future research should consider intervention-based approaches to determine whether targeted physical or tactical adjustments directly influence scoring or defensive success. Second, the analysis did not incorporate match-state variables such as scoreline or temporal phases of play. These factors are well-known to influence tactical choices and physical demands, and their integration could clarify when specific efforts translate into goal events. For instance, highintensity outputs may at times represent proactive attacking strategies, while in other context they may indicate reactive responses to evolving game situations. Third, no stratification by tactical formation or player position was applied. Given that positional roles impose distinct physical and tactical demands, role-specific modelling could enhance the practical value of the findings for training and match preparation. Although a wide range of physical performance metrics was initially tested, only those with statistically significant associations (*p* < 0.05) were discussed to preserve clarity and focus on the most robust predictors. Moreover, despite the dual-team modelling approach, the study relied on average aggregated opponent metrics across matches. More detailed within-match analyses, capturing intensity, clustering of effort, or the impact of substitutions, may uncover pivotal moments that shift game momentum and better explain performanceoutcomes dynamics.

Finally, although the inclusion of two consecutive seasons strengthens analysis, extending the dataset across different leagues, genders, and age group would improve the generalizability of results. Differences in tactical culture, physical standards, and developmental contexts may lead to varied performance-outcome relationships, thereby enriching the broader understanding of match play demands.

### Practical Applications

The strong association between sprinting distance in-possession and goal-scoring effectiveness suggests that contextual drills reproducing these actions, such as tactical exercises in which players accelerate into space under defensive pressure, should be included in the weekly training routine. These drills integrate technical ball control, tactical decision-making, and HSR under realistic constraints. Moreover, the present findings suggest that excessive use of horizontal or backward ball circulation may hinder the creation of scoring opportunities, as it disrupts offensive momentum and delays progression into threatening areas. Accordingly, training interventions should prioritize forward-oriented actions such as progressive passes, dribbles, and runs with the ball. Drills with scoring systems that reward forward progression and penalize regressive passing can serve as effective methods to promote verticality in possession. Furthermore, tactical exercises should focus on coordinated off-the-ball movement to open forward passing lanes, thereby reducing reliance on sideways or backward play. These strategies may improve players’ decisionmaking under pressure and enhance the team’s ability to sustain attacking momentum. Similarly, the role of AMP_NB_ emphasizes the importance of repeat-effort capacity, particularly in pressing and recovery contexts. Practical applications should therefore emphasize contextual training drills that replicate these dynamics (e.g., pressingfocused games or transition-based scenarios), as they naturally combine metabolic and tactical loads. Conditioning methods such as high-intensity intervals or repeated-sprint training may still be used as complementary strategies, but the emphasis should remain on game-specific formats. At the same time, the presence of both positive and negative associations indicates that high-intensity outputs should not be interpreted in isolation. For example, the negative relationship between H-dec_OP_ and offensive success underlines the need to evaluate physical metrics in their tactical context.

## CONCLUSIONS

Contextualized physical performance indicators, particularly sprinting distance and high metabolic power distance, can modulate offensive and defensive outcomes, whereas high-intensity deceleration distance appears to influence offensive outcomes, all contingent on tactical and situational context. The principal contribution of this study lies in its dual-team and context-sensitive framework, which integrates data from both reference and opponent teams across in-possession, out-of-possession, and out-of-play phases. This approach enables a nuanced evaluation of physical performance, highlighting that the effectiveness of high-intensity actions depends not solely on their magnitude, but on their alignment with tactical demands and match circumstances. These findings provide practical guidance for coaches and analysts, supporting informed decisions aimed at optimizing team performance in elite football.
